# Multiple Myeloma and Paget Disease with Abnormal Skull Lesions and Intracranial Hypertension

**DOI:** 10.4084/MJHID.2012.068

**Published:** 2012-11-05

**Authors:** T. Caravita, A. Siniscalchi, E. Montinaro, R. Bove, M. Zaccagnini, D. De Pascalis, A. Morocutti, L. Brusa, F. Arciprete, ML. Cupini, A. Perrotti, E. Palma, S. Fratoni, R. De Simone, C. Iani, P. de Fabritiis

**Affiliations:** 1 Hematology Department, S. Eugenio Hospital – Rome – Italy; 2 Neurology Department, S. Eugenio Hospital – Rome – Italy; 3 Radiology Department, S. Eugenio Hospital – Rome – Italy; 4 Histology Department, S. Eugenio Hospital – Rome – Italy

## Abstract

We report a 73 years old man with a diagnosis of Paget Disease (PD) and symptomatic Multiple Myeloma (MM). Coexistence of MM and PD has rarely been described. PD mimics many of the features of bone destructive process in MM, making differential diagnosis more complicated. In addition, the presence of serious muscolo-skeletal and metabolic complications in both diseases makes management of patients difficult, worsening the prognosis.

The comparison of these two diseases has led to the characterization of a common molecular mechanism represented by the receptor activator of nuclear factor-kB ligand (RANKL)/Osteoprotegerin signaling pathway. The improved comprehension of these mechanisms led to the development of new pharmacologic agents (bisphosphonates, cytokines inhibitors) effective for the treatment of these bone diseases.

## Case Report

**Figure 1:**
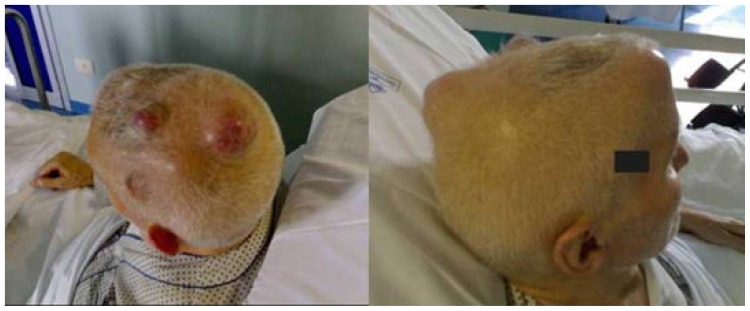
Skull lesions imaging. Presence of skull tumors over the right frontal and parieto-occipital regions.

A 73 years old man with a diagnosis of Paget disease (PD) made in 1978, was admitted in April 2007 to our Neurology Department for a left sided motor deficit and headache. Since the last three years he was suffering of vertebral fractures developing marked dorsal column kyphosis and, in the last three months, he suffered from asthenia, headache and noticed the progressive growth of skull tumors over the right frontal and parieto-occipital regions (Figure 1).

Relevant laboratory findings were: erythrosedimentation rate (94 mm/h), alcaline phosphatase (1500 mU/ml), β2 microglobulin (2380 μgr/L), lactate (570 mU/ml), monoclonal Bence-Jones protein of λ-type and a monoclonal band IgA/ λ-type on serum electrophoresis (Table 1). Blood cell count and serum calcium were normal.

**Figure 2:**
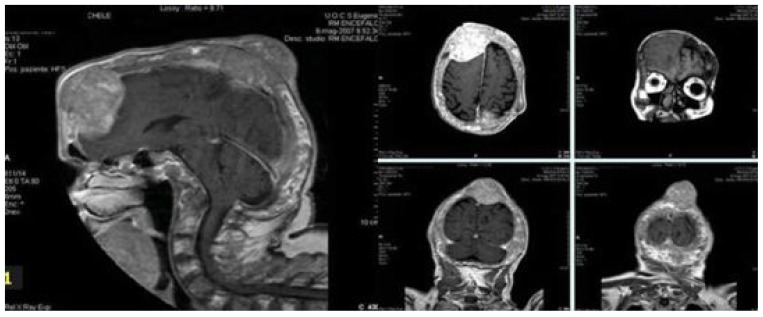
Cranial MRI. Evident eso-endophytic mass lesions through the skull with contrast enhancement and brain parenchima compression.

Osmotic therapy with infusion of 20% hyperosmolar mannitol solutions and steroid therapy promptly relieved symptoms. The patient underwent X-ray, CT scan and MRI brain scan which showed evident eso-endophytic mass lesions through the skull with contrast enhancement and brain parenchyma compression (Figure 2) along with Pagetic bone features.^1^,^2^ Because of the poor compliance, CSF was contra-indicated. Skull lesion biopsy showed a mixed tapetum of monoclonal plasmocitoid elements with a positive immunostaining for λ-light chains. Bone marrow biopsy revealed coexistence of both mature plasma cells with bone remodelling due to increased osteoclastic activity and osteoblastic hyperplasia consistent with Paget disease (PD) and CD138 positive plasmacells of monoclonal λ-light chains type, consistent with multiple myeloma (MM).

Because of the symptomatic MM, cyclophosphamide infusion at 1000 mg/daily dose, days 1, 4 plus Dexamethasone 40 mg/die days 1–4 was started after cranial radiotherapy. A rapid recovery from symptoms and disappearance of skull masses were observed. The patient survived with symptoms relief for four months.

Coexistence of MM and PD has rarely been described.^3^,^4^ MM is a neoplastic plasma-cell disorder characterized by clonal proliferation of malignant plasma cells in the bone marrow microenvironment, monoclonal protein in the blood or urine, and associated organ dysfunction (hypercalcemia, renal insufficiency, anemia, or bone lesions). It accounts for approximately 1% of neoplastic diseases and 13% of hematologic cancers.^5^ The most common symptoms on presentation are fatigue, bone pain, and infections.

Neurologic complications have been reported in 10 to 40% of patients, sometimes as first manifestation of the disease. Indication for treatment in symptomatic myeloma is defined by current guidelines.^6^

PD is a chronic disease of the skeleton featuring one or more areas of aggressive osteoclast-mediated bone resorption preceding imperfect osteoblast-mediated bone repair. The following deranged skeletal remodeling causes bone expansion and softening, becoming unnaturally deformed with pain, fracture and, rarely, neoplastic transformation. Diagnosis is characterized by elevation of serum alkaline phosphatase and characteristic X-ray features. Neurologic symptoms are not frequent. The treatment is directed toward controlling the disease activity and the management of its complications. Bisphosphonates (BPs), both oral and intravenous, have improved Paget treatment reducing and normalizing bone turnover, as measured by biochemical markers and by clinical improvement.^7^ The most devastating complication is osteosarcoma in about 1% of cases.^1^ PD mimics many of the features of bone destructive process in MM, making differential diagnosis more complicated. The radiographic features of PD vary with the stage of the disease: osteolysis dominate early stage as radiolucent lesions; apposition of “Paget bone” creates islands of density called “cotton wool” in the middle stage, and bone becomes homogeneously dense for the osteoblastic apposition in the late stage. In MM the characteristic lesions are generally represented by “punched-out” 1–4 cm radiolucent lesions. Although radiography usually is sufficient for enabling the diagnosis of PD, occasionally a differential diagnosis of sclerotic or lytic metastases needs to be considered. In these cases, computerised tomography (CT) or magnetic resonance imaging (MRI) is generally diagnostic.^8^ The management of patients with PD and symptomatic MM may be more difficult due to the presence of more serious complications, finally worsening the prognosis.

The osteoclast represents the crucial cell involved both in the pathogenesis of PD and bone lysis of MM. The comparison of these two diseases has led to the characterization of a common molecular mechanism represented by the receptor activator of nuclear factor-κB ligand (RANKL)/Osteoprotegerin signaling pathway. It normally drives the physiologic balance between bone resorption/deposition in the bone microenvironment of both diseases, disturbed by the altered trafficking of immune cells and cytokines.^9^,^10^ The improved comprehension of these mechanisms led to the development of new pharmacologic agents (bisphosphonates, cytokines inhibitors) effective for the treatment of these bone diseases.

## Figures and Tables

**Table 1:** Exams at presentation.

Erythrosedimentation rate	94 mm/h
Alcaline Phosphatase	1500 mU/ml
β2 microglobulin	2380 μg/L
Lactate	570 mU/ml
Bence-Jones protein	λ-type
Monoclonal component	IgA/ λ-type
Skull lesion biopsy	mixed tapetum of monoclonal plasmocitoid elements with a positive immunostaining for λ-light chains
Bone marrow biopsy	coexistence of bone remodelling due to increased osteoclastic activity and osteoblastic hyperplasia and CD138 positive plasmacells of monoclonal λ-light chains type
